# Prevalence of Hepatitis C Virus Among Hospitalized Patients in a Tertiary Hospital in Italy: The Basis for a National Screening Assessment Model?

**DOI:** 10.3390/microorganisms13010129

**Published:** 2025-01-10

**Authors:** Giulia Morsica, Massimo Locatelli, Gema Hernandez-Ibarburu, Francesca Rusconi, Alba Segovia-Hilara, Davide Di Napoli, Matteo Moro, Salvatore Mazzitelli, Hamid Hasson, Federico Esposti, Roberts Mazzuconi, Antonella Castagna

**Affiliations:** 1 Infectious Diseases Unit, IRCCS San Raffaele Scientific Institute, 20132 Milan, Italy; hasson.hamid@hsr.it (H.H.); castagna.antonella@hsr.it (A.C.); 2 Laboratory Medicine Service, IRCCS San Raffaele Scientific Institute, 20132 Milan, Italy; massimo.locatelli@hsr.it; 3 TriNetX Europe NV, Kortrijksesteenweg 214 b3, 9830 Sint-Martens-Latem, Belgium; gema.hernandez@trinetx.com (G.H.-I.); francesca.rusconi@trinetx.com (F.R.); alba.segoviahilara@trinetx.com (A.S.-H.); 4 Infection Control, Medical Office, IRCCS San Raffaele Scientific Institute, 20132 Milan, Italy; davide.dinapoli@hsr.it (D.D.N.); moro.matteo@hsr.it (M.M.); mazzitelli.salvatore@hsr.it (S.M.); mazzuconi.roberts@hsr.it (R.M.); 5 Transformation Office, IRCCS San Raffaele Scientific Institute, 20132 Milan, Italy; esposti.federico@hsr.it; 6 Infectious Diseases Unit, Vita-Salute San Raffaele University, 20132 Milan, Italy

**Keywords:** HCV, antibodies, in-hospital screening, birth cohort, prevalence

## Abstract

Free-of-charge hepatitis C virus antibody (HCV Ab) screening in some key populations and in 1969–1989 birth cohorts have been funded in Italy as the first step in confirming diagnosis in individuals who may be unaware of their infection. The purpose of this study is to leverage existing in-hospital routine screening data to better understand the distribution of HCV. A retrospective study of hospitalized patients (PTs) tested for HCV Ab for 5 years (from January 2017 to December 2022) in San Raffaele hospital was conducted according to age categories: birth year group before 1947 (patients older than 76 years old), birth year group 1947–1968, birth year group 1969–1989, and two other groups with birth year groups 1990–2000 and 2001–2022 (with patients younger than 33 years old) using the TriNetX platform. Among the 42,805 in-hospital PTs tested, 1297 (3.03%) were HCV Ab positive. The prevalence of HCV Ab was greater in PTs over the age of 76 (5.3%), whereas it was lower in the youngest birth year cohort (2000–2022, 0.16%). Among 1297 HCV Ab positive PTs, only 198 (15.3%) were tested for the presence of HCV RNA. The birth cohort 1969–1989 had a modest seroprevalence (1.5%), yet they were the most affected age group, with 44.4% being HCV RNA positive. The in-hospital HCV screening including birth year cohort 1947–1989 could be a more valuable option compared to the screening for birth year group 1969–1989 in the general population.

## 1. Introduction

Chronic hepatitis C virus (HCV) infection is a serious public-health problem, with an estimated global prevalence of 0.7% (0.7–0.9%), accounting for roughly 56.8 million HCV infections [1]. Over the past two decades, 48% of global hepatitis-related mortality is attributable to hepatitis C virus infection [2].

Hepatitis C virus infection has caused 48% of global hepatitis-related deaths during the last two decades [2]. The estimation of people with HCV chronic active infection in Italy in 2020 comprised roughly 280,000 persons who were potentially asymptomatic, and furthermore about 120,000 persons with advanced liver fibrosis/cirrhosis with an uncured HCV infection [3]. By 2030, the World Health Organization set the following goals for all hepatitis viruses: 90% fewer cases, 65% fewer deaths, 90% of chronic cases diagnosed, and 80% of people with chronic hepatitis receiving treatment [4]. To achieve the HCV elimination goal by 2030, the general-population screening of 1948–1988 birth cohorts and of those with risk factors for HCV infection independent of their age has been recommended in Italy [5,6]. Very recently, free-of-charge HCV screening in some key populations and in 1969–1989 birth cohorts have been funded in Italy as the first step to diagnosing individuals who are infected but asymptomatic (https://www.salute.gov.it/, accessed on 3 May 2024). Preliminary data in Lombardy showed 0.58% of HCV seroprevalence by screening about 170,000 people in the birth group 1969–1989, representing about 5% of the overall targeted population (https://www.networkhand-hcv.it/, accessed on 3 May 2024).

We examined the seroprevalence for HCV by extracting data from in-hospital HCV screening in a tertiary hospital in Milan, Lombardy, since a customized roadmap for each region is crucial to achieving the high effectiveness of this intervention nationwide. The decision-making process at the regional level to define screening and linkage-to-care may benefit from our findings.

## 2. Materials and Methods

### 2.1. Study Design

This analysis is a non-interventional, retrospective study conducted with data obtained from TriNetX, LLC, Cambridge, MA, USA (“TriNetX”). TriNetX is a global federated health research network providing access to electronic medical records (EMRs) from healthcare organizations (HCOs) worldwide. Once data are sent to the network, they are mapped to a standard and controlled set of clinical terminologies and undergo a data quality assessment including “data cleaning” that rejects records which do not meet the TriNetX quality standards. The TriNetX database performs an internal and extensive data quality assessment with every refresh based on conformance, completeness, and plausibility. Available data types within the network include demographics, diagnoses (represented by ICD-10-CM codes), procedures (coded in ICD-10-PCS or CPT), and measurements (coded to LOINC) from approximately six hundred thousand patients from the San Raffaele hospital healthcare organization. All data collection, processing, and transmission were conducted in compliance with all Data Protection laws applicable to the contributing HCOs, including the EU Data Protection Law Regulation 2016/679, the General Data Protection Regulation on the protection of natural persons regarding the processing of personal data and the Health Insurance Portability and Accountability Act, and the US federal law which protects the privacy and security of healthcare data. The San Raffaele Hospital Network is a distributed network, and analytics were performed at the HCO with only aggregate results being returned to the platform. Individual personal data did not leave the HCO. TriNetX is ISO/IEC 27001:2022 [7 August 2024, TriNetX LLC, Cambridge, MA, USA] certified and maintains a robust IT security program that protects both personal data and healthcare data [7]. The analyses were generated with TriNetX platform software (www.live.trinetx.com, accessed on 10 May 2023) (TriNetX, Cambridge, MA, USA) and patients included in the study were selected from the network at San Raffaele hospital on 10 May 2023. We defined the TriNetX cohort patients hospitalized between 2017 and 2022 by the positivity of HCV antibodies (HCV Ab+) identified with the LOINC code 16128-1 “Hepatitis C Ab [Presence] in Serum” within a month since hospitalization; HCV Ab positivity by enzyme immune assay (EIA, COBAS AMPLICOR, Version 2, Roche Diagnostics S.p.A., Monza, Italy) identified with LOINC code 16128-1 “Hepatitis C Ab [Presence] in Serum” was available in electronic medical records only if confirmed by using the INNO-LIA HCV score line immunoassay (Fujirebio, Europe N.V., Gent, Belgium). The positivity of HCV-RNA testing was identified using the LOINC code 11259-9 “Hepatitis C virus RNA [Presence] in Serum or Plasma” by nucleic acid amplification (NAA, with probe detection, limit of detection 10 IU/mL, COBAS AMPLICOR, Hepatitis C Virus Test version 2.0, Roche Diagnostics S.p.A, Monza, Italy). Patients with an HCV infection diagnosis identified with ICD-10-CM codes B17.1 “Acute hepatitis C”, B18.2 “Chronic viral hepatitis C”, and B19.20 “Unspecified viral hepatitis C without hepatic coma”, together with the positive HCVRNA testing before the screening, were excluded from the analysis. Sub-cohorts of PTs were created based on the year of birth, with the following birth of year ranges: patients born before 1947, born in 1947–1968, born in 1969–1989, born in 1990–2000, and born in 2001–2022. We chose to investigate the prevalence of HCV Ab by age of birth since the graded screening approach that incorporates age of birth is the most cost-effective when compared to models that solely target at-risk individuals or other birth cohorts [5]. According to earlier research conducted in Italy, the prevalence of HCV Ab positive rose with age, particularly among the elderly.

Biochemistry included liver enzymes [aspartate aminotransferase, AST (normal values < 35 IU/L); alanine aminotransferase, ALT (normal value < 59 IU/L); gamma glutamyl transpeptidase, GGT (normal value < 68 IU/L)]; platelets count (normal value 130–400 × 10^9^/L); total bilirubin (normal value < 1.1 mg/dL); and alkaline phosphatase, ALP (normal value < 129 IU/L). These laboratory analyses reflecting necro-inflammatory activity and liver function were selected to better characterize the study cohort.

Since this study used only de-identified patient records and did not involve collection, use, or transmittal of individually identifiable data, this study was exempt from the Institutional Review board approval [8]. Informed consent was not required. This study was conducted adhering to the principles of the Declaration of Helsinki.

### 2.2. Statistical Analysis

All analyses were generated using TriNetX Platform (TriNetX, Cambridge, MA, USA) and analytics were performed on pseudonymized data housed at the HCOs, with only aggregate results being returned to the TriNetX Platform. During the integration of the hospital data intro TriNetX, the clinical history number of the patients was pseudonymized. In Article 4 of the GDPR (https://gdpr-info.eu/, accessed 3 October 2024), the process of pseudonymization is defined as “the processing of personal data in such a manner that the personal data can no longer be attributed to a specific data subject without the use of additional information provided that such additional information is kept separately and is subject to technical and organizational measures to ensure that the personal data are not attributed to an identified or identifiable natural person”. Pseudonymization is a method that allows you to switch the original data set with an alias or pseudonym. It is a reversible process that de-identifies data but allows the re-identification later if necessary. This pseudonymized ID is provided to TriNetX and it is what was used to identify different patients, following the GDPR standards.

Demographics information of the cohorts included age and sex (male and female). Continuous variables were expressed as mean values and standard deviation, categorial data were represented as frequencies and percentages. An incidence analysis was used to calculate the HCV Ab positivity among the tested PTs over the years, from 2017 to 2022. Incidence proportion calculation method in TriNetX assumed an analysis ran on EHR-data [9]. For a given time window, the incidence proportion denominator (PTs at risk) included the PTs of the cohort whose fact records overlapped the time window by at least one day and whose fact records did not contain the event of interest before the given time window. The incidence numerator (incident cases) included all those PTs who were in the denominator and whose records included the event of interest on a date within the time window.

## 3. Results

**Figure 1:**
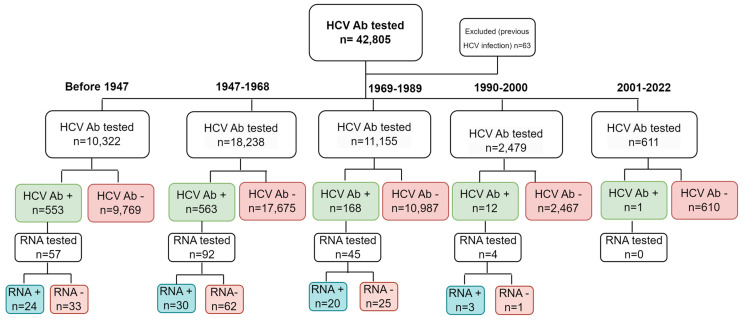
Study design and flowchart of the study cohort. A total of 42,805 PTs was considered in the analysis and divided into 5 classes (born before 1947, born in 1947–1968, born in 1969–1989, born in 1990–2000, and born in 2001–2022). HCV Ab+ and HCV Ab− refer to HCV Ab positivity and negativity, respectively; RNA+ and RNA− refer to HCV RNA positivity and negativity, respectively; n refers to the number of PTs tested.

A total of 181,869 PTs was hospitalized from January 2017 to the end of December 2022 at the Scientific Institute San Raffaele, Milan, Italy. During this time frame, a total of 42,868 were HCV Ab tested. Among these, 63 had a previous diagnosis of HCV infection and were excluded from the analysis. As a result, 42,805 PTs in total were considered for the analysis. Figure 1 displays the study cohort’s flowchart.

Characteristics of PTs according to the birth year cohort are described in Table 1. In general, PTs were more likely to be male (52% of males vs. 48% of females). Concerning liver enzymes, mean aspartate aminotransferase (AST) levels were found slightly increased in the study cohort (including all birth year cohorts) but all other biochemical parameters consistently fell within the normal range.

**Figure 2:**
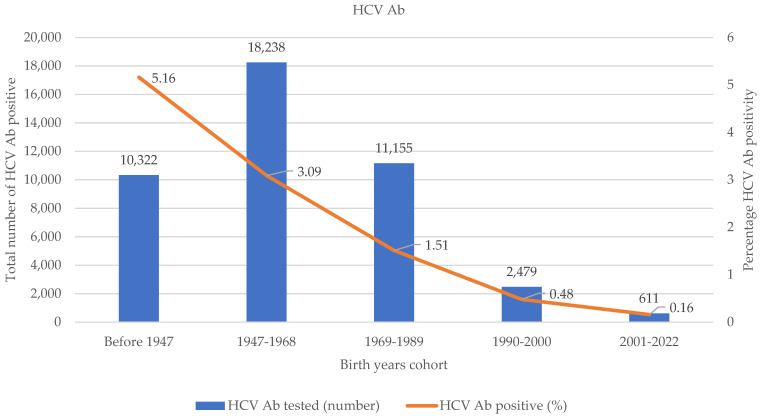
HCV Ab prevalence in the study cohorts during the period 2017–2022. Blue bars denote the number of PTs tested for HCV Ab and red line denotes the percentage of HCV Ab positive among tested PTs.

Among 42,805 PTs tested for the presence of HCV Ab, 1297 (3.03%) were found HCV Ab positive. The prevalence of HCV Ab decreased from older to younger age with a higher proportion (5.1%) in PTs older than 76 years and the lower prevalence in birth cohort 2001–2022 (0.16%) (Figure 2).

Table 2 details the total number of PTs tested for HCV Ab, incident cases, incidence proportion by sex (male and female), and HCV Ab assessment year (2017–2022). The incidence of seroprevalence was higher in men (3.5%) compared to women (2.67%). However, the incidence of seroprevalence decreased in males as well as females during the period of observation, a part of year 2021, where a slight increase IN HCV Ab was revealed in both groups.

Characteristics of PTs with HCV Ab positivity including biochemical parameters are described in Table 3.

The male sex was prevalent in all age cohorts considered. Concerning liver enzymes, mean AST levels were higher than normal values in all age groups, while ALT levels were found higher than normal values only in birth cohort 1969–1989; GGT values were abnormally high in age cohorts 1947–1968 and 1969–1989, and total bilirubin was slightly increased in age cohort 1969–1989.

The prevalence of an active infection assessed by HCV RNA positivity in HCV Ab positive PTs, according to birth year cohort, is depicted in Table 4. Among 1297 HCV Ab positive PTs, only 198 (15.3%) were tested for the presence of HCV RNA. Among 198 HCV Ab positive PTs tested for HCV RNA, an active infection was found in 38.8%. The highest rate of 75% was detected in the 1990–2000 birth year cohort. However, only four PTs in this age group were investigated for the presence of HCV RNA. In the birth year cohort 1969–1989, 44% of PTs had active HCV infection, while 42.1% of those PTs older than 76 years (before the 1947 birth year group) and 32.6% in the 1947–1968 birth year group showed evidence of HCV active infection.

When looking at the characteristics of PTs with active HCV infections, it was found that 68% of them were male. Mean AST, ALT, and GGT levels were found higher than normal values (AST, about 2.5-fold higher than the normal levels; ALT and GGT about 2- and 1.5-fold higher than the normal levels, respectively). Bilirubin levels were slightly increased. The other parameters were within the normal range.

## 4. Discussion and Conclusions

In the present study, we showed that in-hospital screening including people born in the years 1947–1989 seems more effective than opportunistic screening in the general population targeted in the birth cohort 1969–1989. Our data showed a cumulative prevalence of HCV Ab of 3.03% measured over 5 years in PTs hospitalized for other comorbidities, with a higher prevalence in older age groups, particularly in PTs older than 76 years (5.3%) and in birth cohort 1947-68 (3.1%). The prevalence was lower in younger PTs with 1.5% in birth group 1969–1989 that dramatically decreased in PTs younger than 33 years, confirming the decreasing HCV infection risk in younger populations in Italy, known as the cohort effect [10,11,12].

Considering the regional distribution of HCV Ab in Italy, it has been recently estimated that nearly 48,000 individuals living in Lombardy are HCV Ab positive, corresponding to a seroprevalence of approximately 0.48% [13,14].

In a prior study by Kondili and colleagues [14], a mathematical model was employed to estimate HCV seroprevalence in Lombardy. Their results indicated a prevalence approximately 2.65% lower than that observed in the present study. According to their birth cohort analysis, individuals aged 51–70 years exhibited a seroprevalence of about 0.07%. By contrast, in our analysis, which focused on individuals aged 54–75 years, the seroprevalence was markedly higher at 3.12%. Similarly, for older age groups, Kondili et al. [14] reported a seroprevalence of approximately 0.04% in individuals aged 71–100 years, which remains significantly lower than the prevalence we observed in our older cohorts.

These discrepancies are likely attributable to differences in the populations analyzed. The mathematical model used by Kondili et al. [14] may have targeted a general population, whereas our study likely included a subset with higher risk factors, such as individuals of lower socioeconomic status and immigrants hospitalized for unrelated reasons. Although we did not specifically assess economic status or the hospitalization rates of immigrants, the San Raffaele hospital serves as a tertiary referral center. It receives patients not only from the metropolitan and peripheral areas of Lombardy but also from other regions of Italy, potentially resulting in a higher observed prevalence of HCV infection.

We also assessed the incidence of HCV Ab during five years of observation, showing an incidence proportion of 2.2% in 2017 that progressively decreased during 2018–2022, confirming that a large proportion of people in Lombardy have already undergone HCV testing and commenced new efficient and safe therapies against hepatitis C. Interestingly, we observed a small wave of HCV Ab positivity in 2021. It is possible that this small increase in the rate of HCV seroprevalence was consequent to the hospitalization of older PTs and/or people suffering other comorbidities during the COVID-19 pandemic. Hepatic manifestations are the common consequences of COVID-19 and PTs with chronic liver diseases are at a higher risk for severe disease and death from COVID-19 [15,16]. Therefore, we hypothesized that a special vigilance was adopted during the COVID-19 pandemic to screen and treat COVID-19-associated liver injury. To the contrary, in 2022 a significant decrease in seroprevalence was observed (0.36%). We do not have a clear explanation for this result. It could be the consequence of a decline in testing volumes adopted in our hospital, rather than a true epidemiological finding. Regarding biochemistry, we did not find liver enzyme alteration in the initial cohort of 42,805 PTs tested for HCV Ab, while in the HCV Ab positive cohort, alterations in transaminases were detected in some birth year cohorts. In all age groups, apart from those younger than 23 years, AST levels were abnormally high, while ALT levels were altered in the birth year cohort 1969–1989. Unfortunately, information on alcohol intake that could be responsible for an increase in AST compared to ALT was not available in our cohort. Finally, altered transaminases were found in PTs with active HCV infection indicating liver damage, despite the different reasons for hospital admission (Table 3).

Previous studies showed that the age of the target population to be screened and prevalence were main drivers of cost-effectiveness [17,18]. Kondili et al. showed that hepatitis C virus screening strategies by birth cohort in the general population in Italy may incur direct medical costs of EUR 6 billion by 2031 [5]. In the present study, we showed that hospitals are an appropriate setting for screening PTs unaware of their HCV status. However, we did not calculate the costs of such a screening.

We hypothesized that the in-hospital screening could be cost-effective compared to opportunistic screening in the general population, firstly because we identified a higher seroprevalence, and secondly because unlike previous reports, our study involved in-hospital human resources, and HCV screening was included within routine biochemical tests [19,20,21]. Unfortunately, only a small subset of HCV Ab positive PTs (198 of 1293, 15.31%) were tested for the presence of HCV RNA, showing active infection in 38.88% of subjects. This result was similar to findings by Piazzolla and colleagues in South Italy, who reported 32% active infection among 356 hospitalized PTs found to be HCV Ab positive [22]. Another report by Messina and colleagues showed 34% HCV active infection in PTs hospitalized for other reasons, with most of them unaware of their HCV status [23].

Several limitations should be acknowledged when interpreting these results. Caution is needed when drawing causal conclusions given the retrospective and observational nature of this study that is associated with unmeasured bias. TriNetX is an online research platform that is potential subjected to entry errors and data gaps. Some physicians may not have included a diagnostic code for having HCV Ab positive and waited until RNA results were available. These patients may have been excluded when those “with acute/chronic hepatitis diagnosis codes and positive RNA diagnosis codes” were omitted. Another important limitation is the lack of screening strategy to identify patients at risk of HCV infection such as intravenous drug use, transfusion, immigration, or elevated ALT values. Hepatitis C virus antibody testing was arbitrarily chosen. Although data from other hospital experiences in Southern Italy yielded similar results, extensive studies, possibly including the implementation of a screening strategy, should be conducted to confirm the results of in-hospital screening as a useful tool for identifying people with an active HCV infection.

In conclusion, the HCV Ab screening including at least birth cohort 1947–1969 could be of interest in hospitalized PTs. The proportion of HCV Ab positive PTs was high in older birth groups, but active infection was higher in people born in 1969–1989, representing working individuals and rendering the probability of transmission higher. Since a high proportion of PTs were not tested for HCV RNA, introducing in-hospital reflex testing (simultaneous HCV Ab and HCV RNA testing that allows one-step confirmation of the HCV active infection), at least in PTs with altered transaminases, followed by linkage to care, could constitute a suitable micro-elimination strategy in Italy and possibly in other countries with similar HCV prevalence.

## Figures and Tables

**Table 1:** Characteristics of the cohort of patients tested for the presence of HCV Ab.

	Overall Patients	Birth Year Cohort				
Characteristics		Before 1947	1947–1968	1969–1989	1990–2000	2001–2022
Number	42,805	10,322	18,238	11,155	2479	611
Age, years	57.8 (±18)	78.9 (±4.7)	62.5 (±6.46)	40.1 (±6.29)	25.6 (±3.45)	12.1 (±6.62)
Males	22,141 (52)	6179 (60)	11,103 (61)	3856 (35)	712 (29)	291 (48)
AST levels, IU/L	50.1 (±373)	50 (±324)	52.9 (±422)	43.4 (±219)	39.6 (±201)	56.4 (±228)
ALT levels, IU/L	44.3 ± 197	38.1 (±158)	42.3 (±167)	49.2 (±230)	42.2 (±166)	59.7 (±163)
GGT levels, IU/L	61.6 (±149)	58.8 (±124)	65.9 (±157)	55.5 (±123)	36.9 (±65.7)	32.9 (±64.1)
PLTs count, ×10^9^/L	232 (±84.8)	218 (±85.8)	233 (±85.3)	242 (±80.3)	239 (±78.6)	268 (±119)
Total bilirubin, mg/dL	0.88 (±1.69)	0.912 (±1.76)	0.975 (±2.02)	0.754 (±1.35)	0.638 (±0.601)	0.573 (±0.702)
ALP levels, IU/L	91.4 (±97.4)	92.9 (±102)	95.4 (±108)	78.4 (±74.3)	68 (±47.1)	176 (±109)

Results are described by mean (standard deviation, SD) or frequency (%). Abbreviations: AST = aspartate aminotransferase (normal values <35 IU/L); ALT = alanine aminotransferase (normal value < 59 IU/L); GGT = gamma glutamyl transpeptidase (normal value < 68 IU/L); PLTs = platelets count (normal amount 130–400 × 10^9^/L); total bilirubin (normal value < 1.1 mg/dL); ALP = alkaline phosphatase (normal value < 129 IU/L).

**Table 2:** Incidence of HCV Ab in the cohort, overall and according to sex (males vs. females) from year 2017 to 2022.

	Years					
	2017	2018	2019	2020	2021	2022
Patients at risk, total number	16,468	18,843	19,440	17,278	15,443	9046
Incident case, number	338	282	264	192	186	35
Incidence proportion, (%)	2.05	1.50	1.36	1.11	1.20	0.39
Males, number	8325	9343	9671	8533	7557	4414
Incident cases, number	186	161	153	116	112	21
Incidence proportion (%)	2.23	1.72	1.58	1.36	1.48	0.48
Females, number	8143	9500	9769	8745	7886	4632
Incident cases, number	152	121	111	76	74	14
Incidence proportion (%)	1.87	1.27	1.14	0.87	0.94	0.30

**Table 3:** Characteristics of patients with HCV Ab positivity according to years of birth.

Characteristics		Years of Birth		
	Overall	Before 1947	1947–1968	1969–1989
Number	1284	553	563	168
Age, mean (standard deviation), years	66.7 (±14.5)	79.7 (±4.9)	62.1 (±6.74)	42.7 (±6.09)
Males (number)	749 (57.6)	282 (51)	356 (63.2)	103 (61.3)
AST levels, IU/L	75.7 (±602)	80.9 (±826)	77 (±400)	57.5 (±117)
ALT levels, IU/L	52.3 (±193)	45.2 (±239)	54 (±149)	71.6 (±157)
GGT levels, IU/L	70.3 (±123)	55.4 (±95.5)	83 (±138)	86.5 (±153)
PLTs count, 10^9^/L	196 (±91.3)	186 (±87.1)	195 (±89.5)	225 (±101)
Total bilirubin, mg/dL	1.08 (±2.19)	0.994 (±2.25)	1.13 (±1.94)	1.26 (±2.8)
ALP levels, IU/L	94.7 (±87.8)	89.6 (±57.6)	97.6 (±97.7)	103 (±134)

Demography and biochemical parameters in birth groups 1990–2000 and 2001–2022 (total number = 13) were not extracted due to privacy policies (less than 10 PTs had HCV Ab positivity in each of these two groups). Results are described by mean (standard deviation, SD) or frequency (%). Abbreviations: AST = aspartate aminotransferase (normal values < 35 IU/L); ALT = alanine aminotransferase (normal value < 59 IU/L); GGT = gamma glutamyl transpeptidase (normal value < 68 IU/L); PLTs = platelets count (normal amount 130–400 × 10^9^/L); total bilirubin (normal value < 1.1 mg/dL); ALP = alkaline phosphatase (normal value < 129 IU/L).

**Table 4:** HCV RNA positivity in HCV Ab positive PTs, according to birth year cohort.

Age Group by Years of Birth	HCV RNA Tested (Number)	HCV RNA Positive (Number)	HCV RNA Positive (%)
Before 1947	57	24	42.11
1947–1968	92	30	32.61
1969–1989	45	20	44.44
1990–2000	4	3	75
2001–2022	0	0	-
Total	198	77	

## Data Availability

The original contributions presented in the study are included in the article, further inquiries can be directed to the corresponding authors.

## Abbreviations

HCV Ab	HCV antibody
HCOs	healthcare organizations
EMRs	electronic medical records
PTs	patients
AST	aspartate aminotransferase
ALT	alanine aminotransferase
GGT	gamma glutamyl transpeptidase
PLTs	platelets
ALP	alkaline phosphatase
SD	standard deviation
